# YAP Activation Is Associated with a Worse Prognosis of Poorly Cohesive Gastric Cancer

**DOI:** 10.3390/jpm13091294

**Published:** 2023-08-24

**Authors:** Maria Bencivenga, Lorena Torroni, Mariagiulia Dal Cero, Alberto Quinzii, Camilla Zecchetto, Valeria Merz, Simona Casalino, Francesco Taus, Silvia Pietrobono, Domenico Mangiameli, Federica Filippini, Mariella Alloggio, Claudia Castelli, Mar Iglesias, Manuel Pera, Davide Melisi

**Affiliations:** 1 General and Upper GI Surgery, Department of Surgery, Verona University, 37126 Verona, Italy; maria.bencivenga@univr.it (M.B.);; 2 Unit of Epidemiology and Medical Statistics, Department of Diagnostic and Public Health, University of Verona, 37134 Verona, Italy; 3 Section of Gastrointestinal Surgery, Hospital del Mar, Hospital del Mar Medical Research Institute (IMIM), Department of Surgery, Universitat Autonoma de Barcelona, 08003 Barcelona, Spain; 4 Investigational Cancer Therapeutics Clinical Unit, Azienda Ospedaliera Universitaria Integrata, 37134 Verona, Italy; 5 Digestive Molecular Clinical Oncology Research Unit, Università degli Studi di Verona, 37134 Verona, Italy; 6 Unit of Forensic Medicine, Department of Diagnostics and Public Health, University of Verona, 37134 Verona, Italy; 7 Anatomical Pathology Unit, Azienda Ospedaliera Universitaria Integrata, 37134 Verona, Italy; 8 Department of Pathology, Hospital del Mar Medical Research Institute (IMIM), 08003 Barcelona, Spain

**Keywords:** poorly cohesive, gastric cancer, YAP expression, tumor progression

## Abstract

Poorly cohesive (PC) gastric cancer (GC) is extremely aggressive in progression, and there is an urgent need to identify the molecular pathways involved. We hypothesized the essential role of the RhoA–YAP axis in these mechanisms. The present observational multicenter retrospective study included 133 patients with PC GC treated at two dedicated European surgical centers between 2004 and 2014. YAP nuclear localization was measured by immunohistochemical (IHC) analysis of tissue biopsies. The complete absence of nuclear reactivity was coded as negative expression; we considered “any positive” as low nuclear expression (>0% but <10% of cells) and high nuclear expression (≥10% of cells). Women represented about half of the present series (52%), and the median age was 64 years (p25–p75 range: 53–75). Neoadjuvant and adjuvant treatments were administered to 10% and 54% of the cases, respectively. Extended systemic lymphadenectomy (D2) was the most common (54%). In nearly all cases, the number of retrieved nodes was ≥15, i.e., adequate for tumor staging (94%). An R0 resection was achieved in 80% of the cases. Most patients were pathological T stage 3 and 4 (pT3/pT4 = 79.0%) and pathological N stage 2, 3a, and 3b (pN2/pN3a/pN3b = 47.0%) at the pathological examination. Twenty patients (15%) presented distant metastases. Five-year overall survival (OS) was significantly higher (*p* = 0.029) in patients with negative YAP (46%, 95% CI 31.1–60.0%) than in the other patients (27%, 17.5–38.1%). Moreover, when controlling for sex, age, pT, pN, and percentage of signet ring cells in the multivariable analysis, YAP expression was a significant predictor of OS (HR 2.03, 95% CI: 1.18–3.51, *p* = 0.011). Our results provide new insights into the role of the YAP signaling cascade, as its activation was associated with a worse prognosis in PC GC.

## 1. Introduction

Gastric cancer (GC) is the fifth most frequently diagnosed cancer and the third most common cause of cancer-related deaths worldwide. Almost 1 million new cases of GC are diagnosed globally, causing more than 723,000 deaths yearly [1]. The poor prognosis for patients with GC can mainly be attributed to the high rate of metastatic disease at the time of diagnosis and the limited effectiveness of the available systemic treatments [2].

GC is a heterogeneous disease with diverse molecular and histological subtypes [3,4]. Based on Lauren’s criteria [5], the histologic classification divides GC into intestinal and diffuse types with distinct clinical profiles. The category of the Lauren diffuse GC subtype corresponds to the WHO poorly cohesive (PC) one, which includes the pure signet ring cell (SRC) type and the poorly cohesive carcinoma (PCC-NOS) type according to the number of tumor cells that display the classical feature of signet ring cells (SRCs) [6].

PC GC has a unique behavior, which is typical in younger patients [7]; it shows a better prognosis than other GC subtypes if it is in an early local stage, whereas it becomes a highly aggressive and resistant disease when it progresses through the gastric wall [8,9]. Indeed, locally advanced PC GC represents a challenge for resection, as the margins are often positive due to unsuspected submucosal tumor spread, and nodal metastases are pretty common, requiring extensive lymphadenectomies compared to non-PC subtypes. Moreover, the risk of metachronous peritoneal recurrence is high when PC GC invades the gastric serosa. In this regard, there is an urgent need to identify the molecular pathways involved in the tumor progression of such an aggressive GC subtype.

Recently, genomic studies have demonstrated distinct mutation patterns associated with the different GC subtypes. PC GC is typically genomically stable, and there are no current target therapies for this subtype of gastric cancer. In particular, a high rate of RHOA mutations (14.3%) was found in diffuse-type tumors but not in intestinal ones. Increased expression of RHOA correlates with higher tumor node metastasis (TNM) staging and poorly differentiated histological GC subtype 1 [4,10]. In detail, RhoA is a member of the Rho GTPase family, including Cdc42 and Rac1 [11]. Rho GTPases are important intracellular signaling molecules that regulate cytoskeleton organization, the cell cycle, and motility. In cancer, Rho activity promotes metastasis by disrupting the epithelial layer and inducing extracellular matrix degradation [12]. However, the mechanism by which RhoA activity may promote diffuse gastric cancer tumorigenesis and metastasis has been poorly understood.

The transcriptional regulator Yes-associated protein (YAP) is emerging as a central determinant of malignancy due to its significant role in reprogramming cancer cells into cancer stem cells and sustaining tumor initiation, progression, metastasis, and chemoresistance [13]. Recent studies utilizing diffuse gastric cancer-engineered mouse models and human cells identified a tumor-promoting role for Yes-associated protein and transcriptional coactivator with PDZ-binding motif (YAP-TAZ), which are transcriptional coactivators that interact with transcriptional enhanced associate domain (TEAD) transcription factors to regulate gene expression [14,15,16]. However, the possible role of YAP in human GC has not been defined to date.

Recent studies have highlighted a cross-talk between small GTPase signaling and the Hippo pathway, suggesting that small GTPase signaling is a novel upstream regulatory element that activates the YAP signaling pathway [17]. In particular, RHO/RAC stimulates RHO-associated protein kinase (ROCK) and p21-activated kinase (PAK), which induce LIM kinase-1 (LIMK) activity and inactivate cofilin, resulting in F-actin accumulation [18]. F-actin sequesters angiomotin (AMOT), a YAP inhibitor, and promotes YAP activation and nuclear translocation [19,20]. YAP has recently been discovered to be a central mediator of cellular mechanotransduction. The status of the cytoskeleton represents the primary mechanism controlling YAP activity: a rigid extracellular matrix (ECM) maintains active YAP in the nucleus while more elastic matrices lead to YAP inactivation. This regulation requires RHO GTPase activity but appears to be independent of the Hippo/LATS pathway [17].

Here, we hypothesized the RhoA–YAP axis’s essential role in the PC GC subtype’s aggressiveness. Moreover, the correlation between YAP activation and tumor morphology was evaluated, i.e., SRCs vs. PCC-NOS.

## 2. Materials and Methods

The present observational multicenter retrospective study included 133 patients affected by resectable PC GC and treated at two dedicated European surgical centers (Azienda Ospedaliera Universitaria Integrata, Verona, Italy, and Hospital del Mar, Barcelona, Spain) between 2004 and 2014. The patients were followed up for 72 months. In each center, a multidisciplinary team, including one expert pathologist, re-evaluated all the enrolled cases to confirm the PC histotype and classified the cases according to the number of cells displaying the morphological features of SRCs. Briefly, PC GC was coded into three categories according to a recent classification by a European consensus of experts [21]: (a) SRC type 1, “pure” SRC cancers having ≥90% of SRCs; (b) SRC type 2, PC GC with an SRC component between >10% and <90%; and (c) SRC type 3, PC GC with ≤10% of SRCs. These series were further categorized into two morphological groups: >10% of SRCs and <10% of SRCs. Tumor specimens were fixed in buffered formalin overnight, embedded in paraffin blocks, and sectioned at a thickness of 3 µm. The sections were stained with hematoxylin and eosin for morphological evaluation, and immunohistochemistry was performed using the antibody anti-active YAP1 (rabbit monoclonal, clone EPR19812, dilution 1:2000, Abcam, Cambridge, United Kingdom). Staining was performed in an automated stainer, the Leica Bond-Max (Leica Biosystems, Milan, Italy), using the ‘Bond Polymer Refine Detection’ system, according to the manufacturer’s protocol. Nuclear staining was considered positive, and the staining was graded according to the proportion of neoplastic cells expressing the antibody, the distribution, and the intensity of the positivity, as described previously [22]. The complete absence of nuclear reactivity was coded as negative expression while both low nuclear expression (>0% but <10% of cells) and high nuclear expression (≥10% of cells) were considered positive expression. The nuclear expression of YAP was investigated to examine if it was related to the clinical outcomes of the patients.

### Statistical Analyses

The clinico-demographic parameters were expressed as the frequency and percentage for categorical variables and the median and interquartile range (p25–p75) for non-normally distributed continuous variables. The significance of the difference in the categorical variables between the YAP group expression was evaluated using the non-parametric Fisher’s exact test and the Wilcoxon–Mann–Whitney rank-sum test for the quantitative variables. These expression levels were correlated with clinical endpoints, including disease-related survival (DRS) and overall survival (OS). The survival curves were estimated using the Kaplan–Meier method, and the log-rank test was used to evaluate the significance of the differences among the curves. The impact of YAP activation on overall survival was further investigated by using the Cox regression model, stratifying by the center and controlling for sex, age, pathological stage, YAP expression, and SRC percentage.

The statistical analyses were performed using STATA statistical software, release 17.1 (StataCorp, College Station, TX, USA), and the statistical significance was set at *p* < 0.05.

## 3. Results

### 3.1. Series Description

Women represented about half of the present series (69/133 = 52%), and the median age was 64 years (p25–p75 range: 53–75) with a minimum–maximum range of 29–90 years. Most patients were cT3/cT4 (83%) and cN+ (65%) at the clinical examination while cM+ cases were rare (6%) (Table 1).

Neoadjuvant and adjuvant treatments were administered to 10% and 54% of the cases, respectively (Table 1). In nearly all cases, surgery was performed with curative intent with an R0 resection rate of 80% (106/133). A proportion of the patients had R1 (11.0%) or R2 (9.0%) resection margins. Total gastrectomy was the preferred procedure (51.1%) and D2 was the most common lymphadenectomy (54%) while D1 and D3 were performed in one-third of the patients (26% vs. 20%).

In nearly all cases, the number of retrieved nodes was ≥15, i.e., adequate for tumor staging (125/133 = 94%). Resection was often extended to the gallbladder or other adjacent organs (Table 1).

Also, at the pathological examination, most patients were pT3/pT4 (105/133—78.0%) and pN2/pN3a/pN3b (63/133—48.0%). Twenty patients (15%) presented distant metastases. Most cases showed an intermediate proportion of SRCs of 10–90% (SRC type 2 = 61%) while 27% of the tumors were low in SRCs (SRC type 3 ≤ 10%) (Table 1).

More than half of the patients (n = 75) experienced cancer recurrence, 24% experienced loco-regional recurrence, and 6% experienced distant metastasis; 68 patients died during the 72-month follow-up. One death was treatment-related, thirteen were due to other causes, and two were due to unknown causes, yielding eighty-four deaths (Table 2).

### 3.2. Immunohistochemistry Characterization

**Figure 1:**
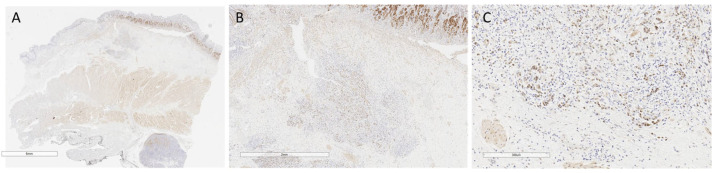
YAP-positive case 1. (**A**) Small-magnification section (6 mm) of YAP AT-stained gastric wall with submucosal infiltrating poorly cohesive cell carcinoma infiltrating the submucosa. (**B**) Detail at higher magnification (2 mm) of neoplastic cells in figure A, with nuclear and cytoplasmic expression of YAP AT involving the submucosa. (**C**) Higher magnification detail (300 µm) of figure B: positive neoplastic cells at the neoplastic advancement front.

**Figure 2:**
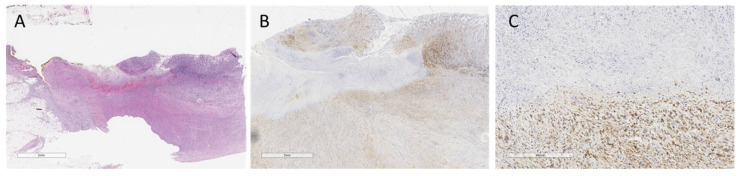
YAP-positive case 2. (**A**) Hematoxylin&Eosin stained section at small magnification (5 mm) of ulcerated gastric wall with poorly cohesive cell carcinoma infiltrating the muscular tonaca propria and adipose tissue. (**B**) Higher magnification detail of A (2 mm): neoplastic cells, with nuclear and cytoplasmic expression of YAP AT involving the muscular tonaca propria. (**C**) Higher magnification detail (300 µm) of B: neoplastic cells, with nuclear and cytoplasmic expression of YAP AT involving the muscle tonaca propria.

In total, 131 cases were suitable for YAP IHC. Most patients were YAP-positive (85/133 = 63.9%) (Table 3). The immunohistochemical analysis of the emblematic YAP-positive cases is reported in Figure 1 and Figure 2.

### 3.3. Association of YAP with Cancer Stage and Prognosis

The clinical tumor stage was significantly earlier in cases that did not exhibit nuclear YAP reactivity. Specifically, about 30% of the YAP-negative cases were at stage cT1–2 while only 10% of the cases showed YAP expression (p = 0.039) (Table 4).

**Figure 3:**
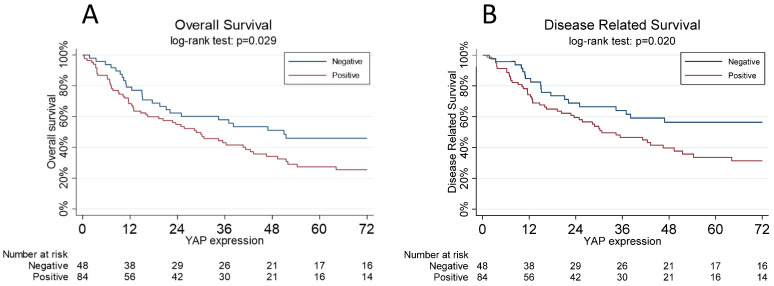
(**A**): The overall survival curves, estimated using the Kaplan–Meier method, are a function of the YAP status: negative vs. positive. One YAP-positive subject was lost at follow-up. (**B**): Disease-related survival, estimated using the Kaplan–Meier method, is a function of YAP status: negative vs. positive. One YAP-positive subject was lost at follow-up.

Five-year overall survival was significantly higher in patients with negative YAP (46%, 95% CI 31.1–60.0%) than in the other patients (27%, 17.5–38.1%, p = 0.029) (Figure 3A).

Moreover, when controlling for sex, age, pT, pN, and percentage of SRCs in the multivariable analysis, YAP expression was a significant predictor of overall survival (HR 2.03, 95% CI: 1.18–3.51, p = 0.011) (Table 5).

The five-year disease-related survival was significantly worse (p = 0.020) in patients with positive YAP (34%, 95% CI 22.3–45.3%) with respect to negative expression (56%, 40.3–67.0%) (Figure 3B).

## 4. Discussion

Poorly cohesive gastric carcinoma is a subtype of GC with increasing relative incidence, mostly in young patients. It has a peculiar behavior: at an early stage, when it is limited to the gastric mucosa or submucosa, it has a better prognosis than all the other GC subtypes, but when it progresses through the gastric wall, it becomes extremely aggressive in most cases. Thus, understanding the molecular mechanisms of tumor progression and biological aggressiveness is of the utmost importance, notably in precision medicine.

The Hippo signaling pathway is a highly conserved potent cell growth, division, and apoptosis regulator. YAP, the nuclear effector of the Hippo pathway, is a key component of this pathway in mammalian systems [22]. There is evidence of a cross-talk between small GTPase signaling and the Hippo pathway during carcinogenesis [17], suggesting an even larger mechanism in which dysfunctions of the junctional complexes and cytoskeletal networks could activate YAP signaling independently of the Hippo/LATS pathway. Moreover, the YAP/TAZ pathway has been demonstrated to be involved in the control of E-cadherin expression/function and thus in the regulation of EMT [23]. In particular, YAP, interacting with Wilms’ tumor protein (WT1), negatively controls E-cadherin expression, which in turn negatively regulates YAP (double-negative feedback loop). In parallel, YAP controls and, though a feedback loop, is controlled by Rac1, a small Rho family GTPase involved in the control of cell migration, mainly through Merlin.

The present study explored the YAP axis in PC gastric tumors by evaluating YAP nuclear expression in a series of 131 PC cases that were highly selected based on the histopathological subtype according to the more recent WHO classification [6]. Remarkably, the nuclear expression of YAP was shown to be an independent factor of a poor prognosis in the present series, suggesting the involvement of this pathway in the progression and acquisition of biological aggressiveness in PC gastric carcinoma.

Altered expression of YAP and its interactors was especially observed at the leading edge of tumor cells, making these changes crucial for cell dissemination (Figure 1 and Figure 2).

The most relevant clinical impact of the present findings would be given by the availability of effective YAP inhibitors. In this regard, it must be remembered that YAP protein does not contain intrinsic DNA-binding domains. Thus, YAP binds to target gene promoters by interacting with DNA-binding transcription factors, such as TEADs. After forming a YAP–TEAD complex, it triggers the transcription of the downstream gene.

Therefore, the most effective mechanism that inhibits YAP is the disruption of such a YAP–TEAD complex. The most representative drugs acting through this mechanism are Veteprofin, CA3, and Super-TDU [24,25].

Veterporfin is the most widely used YAP inhibitor and is FDA approved in combination with light for the photodynamic treatment of neovascular macular degeneration. More specific and effective inhibitors such as CA3 are still in preclinical phases but will hopefully be available for clinical use in the near future.

Of note, other mechanisms of YAP inhibition have been described [24,25].

The dephosphorylated version of YAP only enters the nucleus and is active as a transcription factor while the unphosphorylated version typically accumulates in the cytoplasm. Pharmacological inhibition of focal adhesion kinase (FAK), a crucial downstream effector in the Hippo pathway, was associated with an increase in the phosphorylated and therefore inactive form of YAP, constituting another promising therapeutic mechanism in this setting [26].

Another interesting finding from multiple studies is that PD-L1 is a direct transcription target of YAP, and thus, YAP activation could upregulate PD-L1 expression and promote tumor immune escape in NSCLC, mesothelioma, and melanoma. Further research is needed to deepen the relationship between YAP and PD-L1 signaling and evaluate the efficacy of YAP inhibition as a mechanism to enhance the efficacy of immunotherapy in gastric cancer [27,28,29,30].

The main limitation of the present study is the small number of patients treated with neoadjuvant chemotherapy: it was indicated in only about 10% of cases in the current series, as neoadjuvant/perioperative treatments were introduced in routine clinical practice for non-cardia gastric cancer starting from 2010. However, the impact of YAP nuclear positivity on chemoresistance should be investigated in a further series.

Moreover, unfortunately, a clear association between YAP activation and tumor morphology, namely the number of signet ring cells, although postulated as a hypothesis, was not observed in the context of the analyzed PC tumors.

We are carrying out studies on animal models to confirm the role of YAP activation as a mechanism of tumor progression in PC tumors of the stomach, and further analyses will concern the relationship between its activation and RohA mutations in this specific clinical subset. However, we believe that the results of the present study are extremely relevant as they demonstrate that with an immunohistochemical investigation, of possible application in daily clinical practice, it is therefore possible to identify those PC tumors which have a worse prognosis and which could benefit from more aggressive treatments and, hopefully, in the immediate future, specific targeted therapies as well.

## 5. Conclusions

In conclusion, our results provide new insights into the role of the YAP signaling cascade as a prognostic and potentially predictive factor in sporadic PC gastric cancer. Further studies will deepen the interconnections between the Hippo pathway and the cytoskeleton dynamics in determining the cell shape, migration, and differentiation of PC tumors.

## Figures and Tables

**Table 1:** Main baseline demographic, clinical, surgical, and pathological patient characteristics.

		Total n = 133 (%)
Sex (women)		69 (52.7)
Age ^1^		64.3 (53–74.5)
cT *	cT1	8 (7.3)
	cT2	10 (9.1)
	cT3	44 (40.0)
	cT4	48 (43.6)
cN *	N0	39 (35.5)
	N+	71 (64.5)
cM *	cM0	103 (93.6)
	cM1	7 (6.4)
Neoadjuvant therapy *	Chemotherapy	13 (10.0)
	Chemo + radiotherapy	1 (0.8)
	No treatment	116 (89.2)
Adjuvant therapy *	Chemotherapy	35 (36.5)
	Radiotherapy	2 (2.1)
	Chemo + radiotherapy	15 (15.6)
	No treatment	44 (45.8)
Gastrectomy	Total	68 (51.1)
	Sub-total	65 (48.9)
Lymphadenectomy	D1	34 (25.6)
	D2	72 (54.1)
	D3	27 (20.3)
Total lymph nodes harvested ^1^		36 (23–45)
Total positive lymph nodes ^1^		7 (0–15)
Resection of adjacent organs (including cholecystectomy)	Yes	94 (70.7)
	No	39 (29.3)
Curativity	R0	106 (79.7)
	R1	15 (11.3)
	R2	12 (9.0)
SRC group *	<10%	34 (26.8)
	10–90%	78 (61.4)
	>90%	15 (11.8)
pT	pT1	21 (15.8)
	pT2	7 (5.3)
	pT3	30 (22.5)
	pT4	75 (56.4)
pN *	pN0	39 (29.8)
	pN1	11 (8.4)
	pN2	18 (13.7)
	pN3	63 (48.1)
pM	pM0	113 (85.0)
	pM1	20 (15.0)
Length of hospital stay (days) ^1^		8 (7–10)

^1^ Median, (p25–p75). cT: clinical stage tumor; cN: clinical stage node; cM: clinical stage metastasis; pT: pathological stage tumor; pN: pathological stage node; pM: pathological stage metastasis; SRC: signet ring cell. * Number of missing data: cT/cN/cM: 23; Neoadjuvant therapy: 3; Adjuvant therapy: 37; SRC group: 6; pN: 2.

**Table 2:** Long-term outcomes in the series under study.

	Total n = 133 (%)
Recurrence ^1^	
Yes	75 (59.2)
No	51 (40.5)
Loco-regional recurrence ^1^	
Yes	24 (24.0)
No	76 (76.0)
Time of recurrence (months) ^2^	11.7 (7.4–24.4)
Distant metastases ^1^	
Yes	8 (6.1)
No	124 (93.9)
Death ^1^	
Yes	84 (67.7)
No	40 (32.3)
Cause of death ^1^	
Therapy-related	1 (1.2)
Cancer-related	68 (82.9)
Other	13 (15.8)

^1^ Information on the overall recurrence, loco-regional recurrence, distant metastases, death, and cause of death was missing in 7, 33, 35, 9, and 2 patients, respectively. ^2^ Median (p25–p75).

**Table 3:** Immunohistochemistry characterization of the series.

	Total n = 133
YAP	
Positive	85 (63.9)
Negative	48 (36.1)
YAP grade	
High	46 (34.6)
Low	39 (29.3)
Negative	48 (36.1)
YAP percentage ^1^	10 (0–20)

^1^ Median, (p25–p75).

**Table 4:** Relation between YAP expression and the main baseline demographic, clinical, and pathological characteristics.

Total n = 133	YAP Expression		*p*-Value
	Negative n = 48 (%)	Positive n = 85 (%)	
Sex			0.104
M	18 (37.5)	44 (53.0)	
F	30 (62.5)	39 (47.0)	
Age ^1^	64 (47–77)	64 (55–73)	0.882
cT			0.039
cT1	4 (10.3)	4 (5.6)	
cT2	7 (18.0)	3 (4.2)	
cT3	16 (41.0)	28 (39.4)	
cT4	12 (30.7)	36 (50.7)	
cN			0.408
cN0	16 (41.0)	23 (32.4)	
cN+	23 (59.0)	48 (67.6)	
cM			1.00
M0	37 (94.9)	66 (92.9)	
M1	2 (5.1)	5 (7.1)	
Neoadjuvant therapy			0.164
Chemotherapy	2 (4.4)	11 (13.1)	
Chemo + radiotherapy	0	1 (1.2)	
No treatment	44 (95.6)	72 (85.7)	
Adjuvant therapy			0.573
Chemotherapy	13 (36.1)	22 (36.7)	
Radiotherapy	0	2 (3.3)	
Chemo + radiotherapy	4 (11.1)	11 (18.3)	
No treatment	19 (52.8)	25 (41.7)	
Gastrectomy			1.00
Total	25 (52.1)	43 (50.6)	
Sub-total	23 (47.9)	42 (49.4)	
Other resection			0.241
Yes	11 (22.9)	28 (32.9)	
No	37 (77.1)	57(67.1)	
Lymphadenectomy			0.544
D1	10 (20.8)	24 (28.4)	
D2	29 (60.4)	43 (50.6)	
D3	9 (18.8)	18 (21.2)	
Total lymph nodes harvested ^1^	33.5 (23–45)	36 (23.0–44)	0.818
Total positive lymph nodes ^1^	6 (0–18)	7 (0–13)	0.856
pT			0.507
1–2	12 (25.0)	16 (18.8)	
3–4	36 (75.0)	69 (81.2)	
pN			0.542
0	16 (34.0)	23 (27.4)	
1–2	8 (17.0)	21 (25.0)	
>3	23 (49.0)	40 (47.6)	
pM			0.801
0	40 (83.3)	73 (85.9)	
1	8 (16.7)	12 (14.1)	
Curativity			0.159
R0	41 (85.4)	65 (76.5)	
R1	2 (4.2)	13 (15.3)	
R2	5 (10.4)	7 (8.2)	
SRC group			0.412
<10%	14 (31.1)	20 (24.4)	
>10%	31 (68.9)	62 (75.6)	
Recurrence			0.256
Yes	23 (52.3)	52 (63.4)	
No	21 (47.7)	30 (36.6)	
Loco-regional recurrence			0.806
Yes	7 (21.9)	17 (25.0)	
No	25 (78.1)	51 (75.0)	
Time of recurrence (months) ^1^	15.8 (9.3–32.2)	10.1 (5.9–20.2)	0.173
Metastases			0.139
Yes	5 (10.4)	3 (3.6)	
No	43 (89.6)	81 (96.4)	
Days of hospitalization ^1^	7.5 (6.5–8.5)	8 (7.0–13.0)	0.157
Death			0.071
Yes	25 (56.8)	59 (73.7)	
No	19 (43.2)	21 (26.3)	
Cause of death			1.00
Therapy-related	0	1 (1.7)	
Cancer-related	19 (82.6)	49 (83.0)	
Other	4 (17.4)	9 (15.3)	

^1^ Median, (p25–p75). cT: clinical stage tumor; cN: clinical stage node; cM: clinical stage metastasis; pT: pathological stage tumor; pN: pathological stage node; pM: pathological stage metastasis; SRC: signet ring cell.

**Table 5:** A multivariable Cox proportional hazard model for the evaluation of the overall survival.

	Haz. Ratio	95% CI	*p*-Value
Sex			
(F vs. M)	0.70	0.43–1.14	0.156
Age	1.03	1.01–1.04	0.001
pT (3–4 vs. 1–2)	3.61	1.44–8.98	0.006
pN			
(1–2 vs. 0)	4.16	1.88–9.16	<0.001
(>3 vs. 0)	3.50	1.70–7.21	0.001
YAP			
(Positive vs. negative)	2.03	1.18–3.51	0.011
SRC			
(≥10% vs. <10%)	0.98	0.55–1.72	0.910

cT: clinical stage tumor; cN clinical stage node; cM: clinical stage metastasis; pT: pathological stage tumor; pN: pathological stage node; pM: pathological stage metastasis; SRC: signet ring cell.

## Data Availability

Upon request, we can share the database from which the work was derived.
